# Breast cancer survivors are at an increased risk for osteoporotic fractures not explained by lower BMD: a retrospective analysis

**DOI:** 10.1038/npjbcancer.2015.10

**Published:** 2015-07-22

**Authors:** Merav Fraenkel, David B Geffen, Victor Novack, Tali Shafat, Yuval Mizrakli, Samuell Ariad, Michael Koretz, Larry Norton, Ethel Siris

**Affiliations:** 1 Endocrine Unit, Soroka University Medical Center and the Faculty of Health Sciences, Ben-Gurion University of the Negev, Beer Sheva, Israel; 2 Department of Oncology, Soroka University Medical Center and the Faculty of Health Sciences, Ben-Gurion University of the Negev, Beer Sheva, Israel; 3 Clinical Research Center, Soroka University Medical Center and the Faculty of Health Sciences, Ben-Gurion University of the Negev, Beer Sheva, Israel; 4 Breast Health Center, Soroka University Medical Center and the Faculty of Health Sciences, Ben-Gurion University of the Negev, Beer Sheva, Israel; 5 Breast Center, Memorial Sloan Kettering Cancer Center, New York, NY, USA; 6 Division of Endocrinology, Columbia University Medical Center, New York, NY, USA

## Abstract

**Background:**

An association between higher bone mineral density (BMD) and the diagnosis of breast cancer (BC) has been reported. Data on the risk of osteoporotic fractures in women with BC are conflicting.

**Aims:**

The objective of this study was to assess fracture risk adjusted for BMD in women with and without BC, and to assess whether fracture risk in BC patients is attributed to BMD or BC characteristics.

**Methods:**

Using electronic medical records of patients who underwent dual energy X-ray absorptiometry BMD studies at Soroka University Medical Center between February 2003 and March 2011, we identified women with subsequent diagnosis of osteoporotic fractures. BC status, demographic, health characteristics, BMD, and other laboratory findings were assessed. In BC patients data on grade, stage, and treatment were collected. Primary outcome was osteoporotic fracture, analyzed by Cox proportional hazards regression models.

**Results:**

During a median follow-up of 4.9 years in 17,110 women with BMD testing (658 BC patients), 1,193 women experienced an osteoporotic fracture (62 in BC and 1,131 in no-BC groups). In multivariate analysis adjusted for age, body mass index (BMI) and BMD, hazard ratio (HR) for any osteoporotic fracture in women with BC was 1.34 (*P*=0.026). BMD was similar among women with and without BC who fractured. BC patients who experienced an osteoporotic fracture had a trend for less-advanced BC, lower rates of chemotherapy treatment, and higher rates of tamoxifen treatment.

**Conclusions:**

BC survivors are at increased risk of an osteoporotic fracture, which is not explained by worse BMD. Chemotherapy or aromatase inhibitors did not contribute substantially to fracture risk among our BC survivors.

## Introduction

Several observational studies have suggested that a higher bone mass is associated with increased breast cancer (BC) risk.^1,2^ However, data on the risk of osteoporotic fractures in women with BC are conflicting. Early studies did not find a lower risk for osteoporotic fracture among patients who developed BC.^3–5^ Large epidemiological studies demonstrated an increased fracture risk in BC patients. In a study comparing fracture rates in postmenopausal women with and without a history of BC in the Women’s Health Initiative cohort, BC patients were at a 15% increased risk of clinical fracture compared with controls.^6^ Furthermore, in the same study, 146,959 postmenopausal women were followed for up to 9 years, and incident BC carried a 55% increase in risk of hip fracture.^7^

Several other contemporary reports demonstrated an unchanged or even lower risk for osteoporotic fractures in BC survivors.^8^ A study conducted in the Mayo Clinic showed no increase in hazard ratio (HR) for an osteoporotic fracture among 608 BC patients.^9^ Two studies from Denmark and the United States each separately reported on lower total and hip fracture risk (respectively) in BC survivors.^10,11^

In view of these conflicting data, we conducted this retrospective study with the primary objective to compare the rates of osteoporotic fractures adjusted for bone mineral density (BMD) in BC patients and controls. We further examined whether fracture risk in BC patients is attributed to BMD or BC treatment.

## Materials and methods

### Population

We identified all women who underwent BMD measurement at Soroka University Medical Center, Beer Sheva, Israel, a 1,000-bed tertiary-care hospital, between January 2003 and March 2011. We excluded those under age 18. Only the initial test was included for women who underwent more than one BMD measurement. The electronic medical records of the patients were assessed. “Clalit” Health Services, the largest of the four health maintenance organizations in Israel, maintains a comprehensive electronic medical record system where the patient is identified by the single national identification number. All medical encounters are recorded in the system.

We screened the electronic medical record for an osteoporotic fracture (fracture of hip, proximal humerus, ribs, spine, or distal radius) diagnosed following the BMD test. Non-osteoporotic, pathologic, and high-velocity trauma fractures were excluded. In addition, the electronic charts were screened for BC diagnosis, using International classification of disease-9 codes (233.0, 174.0–175.9), pathology diagnoses (biopsy), and surgical procedures. For BC patients, oncology department medical charts were manually reviewed to obtain the additional clinical information. Data were collected on demographics, mortality, body mass index (BMI), laboratory results, medication purchase, and BC characteristics (risk factors, histological diagnosis, stage, grade, and treatment modalities). The study protocol was approved by the Soroka Medical Center Institutional Review Board.

### BMD measurement

BMD was measured by dual energy X-ray absorptiometry using a Prodigy densitometer (GE-Lunar, Milwaukee, WI, USA) at the lumbar spine, femur neck, and total hip. The results were expressed as bone density (in g/cm^2^), *T*-score (s.d. from the mean for young women), and *Z*-score (s.d. from the mean for age-matched women adjusted for body mass).

### 25-hydroxyvitamin D assay

Vitamin D status was reported when available. Vitamin D levels were determined by measuring patients’ serum 25-hydroxyvitamin D levels by the IDS Octavia 25-OH-D Kit (Immunodiagnostic Systems, Boldon, UK). Results were expressed as ng/ml with a normal range of 20–58 ng/ml.

### PTH assay

Parathyroid hormone (PTH) status was reported when available. Serum PTH levels were determined by using the Immulite 2000 intact PTH Kit (Siemens, Los Angeles, CA, USA). This determination is based on a solid phase, two-site chemiluminescent enzyme-labeled immunometric assay. Results were expressed as pg/ml with a normal range of 14–72 pg/ml.

### Primary outcome

The primary outcome was osteoporotic fracture defined as fracture of hip, proximal humerus, ribs, spine, or distal radius diagnosed after BMD was performed.

### Statistical analysis

The results are presented as the mean±s.d. for continuous variables, as total patients (percentage of total patients) for categorical data, and median and interquartile range for variables with non-normal distribution. The *t*-test was used for comparison of continuous variables and *χ*^2^- or Fisher’s exact tests were used for categorical data. We utilized the Mann–Whitney test for the comparison of variables with non-normal distribution. Multivariate analyses for osteoporotic fracture risk factors were performed using Cox proportional hazards regression models. Variables found to be associated with the outcome in the univariate analysis with *P* value<0.1 and clinically significant factors were included in the models after verifying the proportionality of the hazards.

A two-tailed *P* value of ⩽0.05 was considered significant. The statistical analysis was done using SPSS version 21 (IBM Corp Armonk, NY, USA).

## Results

### Study population

**Figure 1:**
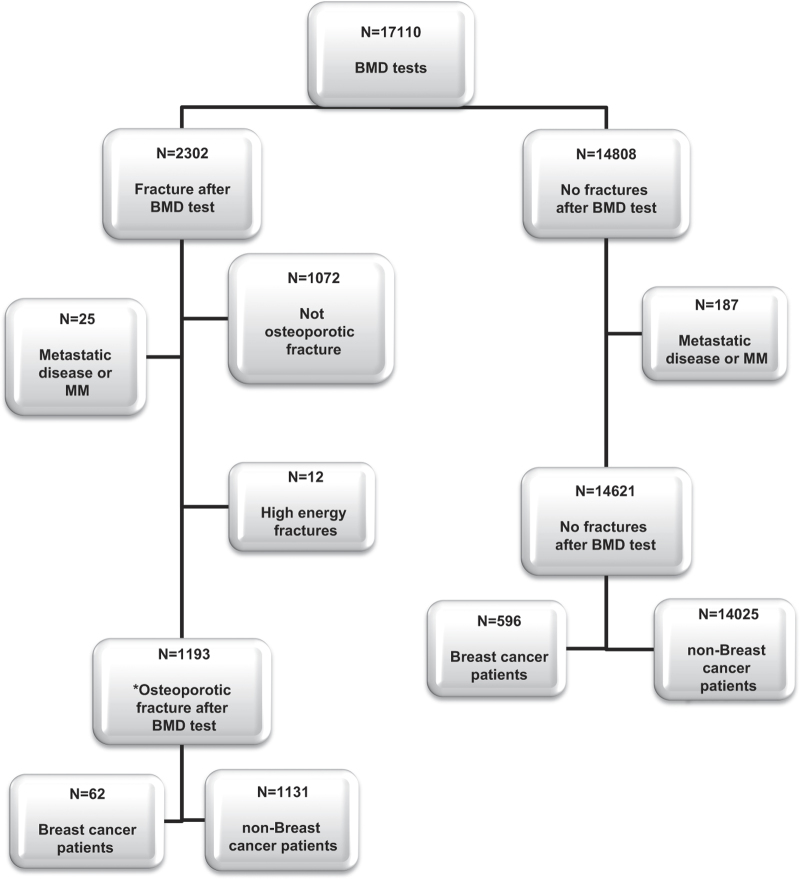
Flow chart of study population. *Osteoporotic fracture defined as hip, vertebral, distal radius, humerus, and ribs fractures.

A total of 17,110 women underwent BMD testing at Soroka University Medical Center between February 2003 and March 2011. Figure 1 presents the study population flow chart. During a median follow-up of 4.9 years, 2,302 women experienced any fracture following BMD, while 14,808 remained free of fracture. In the present study we focused on the 1,193 patients with osteoporotic fractures comprising 62 women with BC and 1,131 BC-free women.

Table 1 presents the baseline characteristics of the women with an osteoporotic fracture according to BC status. Women with and without BC experienced an osteoporotic fracture at a similar age and BMI. Before BMD test women without BC had higher rates of vitamin D and bisphosphonate use as compared with women with BC. BMD at all three sites, lumbar spine, femur neck, and total hip, was similar between women who fractured with and without BC as measured by three methods of assessment: g/cm^2^, *T*-score and *Z*-score.

Table 2 presents fracture characteristics compared between women with and without BC. Anatomic location of osteoporotic fracture did not differ according to BC status. The median time from BMD testing to first osteoporotic fracture did not differ between women with and without BC. Median time from BC diagnosis to BMD testing was 6.2 years (interquartile range 3.3–9.4 years).

### BC patients with and without osteoporotic fractures

Baseline characteristics of BC patients with and without an osteoporotic fracture are presented in Table 3. BC patients who fractured were slightly older than those without fractures, but had similar BMI. Rates of prior usage of hormone replacement therapy and oral contraceptives were not different among BC patients with and without fracture (Table 3a). During study follow-up 71 BC patients died.

### Stage and grade of among BC patients with and without an osteoporotic fracture

Women with fractures had a trend toward less-advanced BC (lower tumor node metastasis stage and grade) compared with those who remained free of fracture (Table 3b). Hormone receptor and Her-2 receptor status by immune-histochemistry did not differ between BC patients who fractured compared with those who did not fracture.

### Treatment for BC and BMD among women with and without an osteoporotic fracture

Data on the BC treatment are presented in Table 3b. A lower percentage of BC patients who fractured as compared with women without fracture received chemotherapy (either adjuvant or neo-adjuvant) for their BC, while a higher percentage received tamoxifen. Rates of aromatase inhibitors use did not differ between BC patients with and without fractures (Table 3b). Rates of radiotherapy and use of trastuzumab did not differ according to fracture status (Table 3b). As expected, BMD in the lumbar spine, femur neck, and total hip expressed as g/cm^2^, *T*- and *Z-*scores were lower among BC patients who fractured compared with those without fracture (Table 3c).

### Factors associated with osteoporotic fracture

We used Cox survival regression models for multivariate analysis of factors associated with osteoporotic fractures (Table 4). Parsimonious model showed that adjusted for age, BMI, and BMD, BC conferred an excess risk for fracture of 34% (HR 1.34, confidence interval 1.04–1.73, *P*=0.026).

## Discussion

Our results show that BC survivors are at 34% increased risk of suffering from an osteoporotic fracture. This increased risk is not explained by worse BMD, as BMD tended to be slightly higher in BC patients who fractured as compared with those without BC who fractured. It is also not explained by higher rates of vitamin D deficiency or use of certain medications that are considered as detrimental for bone health such as systemic or topical steroids or anticonvulsants. Compared with women who fractured but did not suffer from BC, a smaller percentage of women with BC who fractured were treated with vitamin D or bisphosphonates before BMD, which may reflect that these women were not considered at high risk for fractures. The results of our work underscore the importance of appreciating fracture risk in BC survivors and treating them according to the updated osteoporosis/BC guidelines.

In subgroup analysis of BC patients, those who fractured had a trend toward older age, but did not differ from BC women who did not fracture in rates of hormone replacement therapy use. Thus, fracture risk is not explained by lack of estrogen treatment in the menopause. Furthermore, BC women who fractured had a trend of less-aggressive disease, were less frequently treated with chemotherapy or aromatase inhibitors compared with BC women who did not fracture. As expected, BC women who fractured had lower BMD compared with those with BC who did not fracture, which contributed to their fracture risk.

The conclusions that have been reached in our population support other studies that showed higher rates of fracture in BC survivors. This is contrary to the assumption that higher BMD may protect BC patients from osteoporotic fractures.^1,2^ Early reports from Sweden and the United States were not protected from osteoporotic fractures.^3,4^

Osteoporotic fracture risk was assessed in a prospective cohort of women (5.1 years’ follow-up) from the Women’s Health Initiative study;^6^ after adjusting for demographic parameters and various risk factors, BC survivors (*n*=5,298) had a HR of 1.15 for any fracture compared with controls (*n*=80,848). In our cohort the HR for fracture among BC survivors was even higher (1.34), and was not explained by worse BMD in BC patients.

We found that vertebral fractures were the most common osteoporotic fracture in BC survivors. An elevated risk for vertebral fracture was found in a cohort of BC patients from the United Kingdom. This increased risk was related to the diagnosis of BC and to the excessive bone loss secondary to the treatment for BC.^5^ This was not true for BC survivors in our cohort, who experienced an osteoporotic fracture despite having a trend for less-aggressive and less-advanced BC compared with BC survivors who did not fracture. In addition, BC survivors who fractured were less heavily treated with chemotherapy or aromatase inhibitors rather a larger proportion received tamoxifen, which is considered protective for bone health. Similar to our work, fracture risk was assessed in a BC cohort of 608 women treated at the Mayo Clinic.^9^ In this work, a standardized incidence ratio of 0.9 (95% confidence interval 0.7–1.2) for osteoporotic fracture risk in BC patients was found. After adjustment for age, they found that advanced disease (stage III/IV), any chemotherapy, alcoholism, and use of bisphosphonates were risk factors for osteoporotic fractures in BC survivors. It may be that underlying clinical characteristics prompting specific treatments may have been partially responsible for the associated fracture outcomes in this study and in our work (indication bias).

There are several limitations to our study, part of which rely on the retrospective nature of data collection from patients files. The database from which patients with and without BC who fractured was from all women who performed BMD at Soroka Medical Center and does not represent all women with BC who were treated in Soroka Medical Center during the same time period, and there may have been a selection bias in patient referral to BMD. Retrospective nature of the data assessment carried inherent limitations of the data availability: for example, there was the lack of data on estrogen exposure (gravidity, parity, age at menarche and menopause, and more). Finally, the possibility of the selection bias should be considered: that is, women with the history of BC may have a closer medical follow-up leading to the better diagnosis of the osteoporotic fractures.

The studies’ strength is based on the unique structure of Israeli Clalit health insurance system that allows access to a fully computerized medical record system, which maximizes availability of baseline and follow-up data including clinical data, lab workup, and medication use. In addition, Soroka Medical Center is unique in the sense that all BC patients are treated in a single institute with access to patient’s records that cover complete treatment scheme. This is also the first study to the best of our knowledge that correlated fracture risk in BC survivors with BMD data.

## Conclusions

In summary we found that BC survivors are at increased risk for osteoporotic fractures and that this increased risk is not explained by worse BMD compared with women who fractured but did not suffer from BC. There may be a qualitative defect in bone of BC patients that is not apparent with BMD testing similar to other processes that affect bone quality such as diabetes and obesity. More novel qualitative technologies that estimate the trabecular microarchitecture, such as the trabecular bone score, might be useful in assessing the risk fracture for these patients.^12^

We also found that treatment with chemotherapy or aromatase inhibitors did not contribute substantially to fracture risk among our BC survivors. This leads us to believe that lower past estrogen exposure leading to less-aggressive BC and therefore less chemotherapy as well as inherent factors from BC that directly negatively affect bone are the main contributors for increased fragility in a subset of BC patients.

## Figures and Tables

**Table 1:** Baseline characteristics of patients with osteoporotic fracture according to BC diagnosis (*n*=1193)

*Variable*	*BC (*n*=62)*	*No BC (*n*=1,131)*	P *value*
Age at first fracture	68.8 (±8.8)	68.8 (±10.2)	0.969^a^
BMI	29.3 (±4.8)	29.6 (±5.6)	0.735^a^
*BMI*			
⩽30	39 (62.9)	637 (56.3)	0.658^b^
30.1–35	15 (24.2)	309 (27.3)	
35.1–40	7 (11.3)	139 (12.3)	
⩾40.1	1 (1.6)	46 (4.1)	
			
*BMD, g/cm*^*2*^			
Femoral neck	0.77 (±0.11)	0.76 (±0.12)	0.317^a^
Total hip	0.84 (±0.13)	0.82 (±0.13)	0.164^a^
Spine	0.96 (±0.15)	0.95 (±0.16)	0.450^a^
			
*BMD* T-*score*			
Femoral neck	−1.72 (±0.94)	−1.85 (±1.00)	0.330^a^
Total hip	−1.32 (±1.12)	−1.52 (±1.10)	0.169^a^
Spine	−1.80 (±1.23)	−1.94 (±1.36)	0.450^a^
			
*BMD* Z*-score*			
Femoral neck	−0.37 (±0.84)	−0.46 (±0.88)	0.401^a^
Total hip	−0.16 (±1.03)	−0.31 (±0.94)	0.220^a^
Spine	−0.29 (±1.34)	−0.43 (±1.36)	0.425^a^
VitD, ng/ml (*n*=465)	21.4 (±9.9) (*n*=28)	19.9 (±9.7) (*n*=437)	0.431^a^

*VitD, ng/ml*			
<20	14 (50.0)	234 (53.5)	0.715^b^
⩾20	14 (50.0)	203 (46.5)	
PTH, pg/ml (*n*=203)	48.7 (±34.0) (*n*=9)	65.7 (±58.1) (*n*=194)	0.384^a^

*PTH, pg/ml*			
⩽72	6 (66.7)	143 (73.7)	0.640^b^
>72	3 (33.3)	51 (26.3)	
			
*Medication use (*N*, %)*			
Topical steroids (>6 months, during 2 years before BMD)	2 (3.2)	55 (4.9)	0.556^b^
Systemic steroids (>3 months, during 2 years before BMD)	2 (3.2)	96 (8.5)	0.142^b^
Hormone replacement therapy (during 2 years before BMD)	13 (21.0)	244 (21.6)	0.910^b^
Vitamin D (during the last 12 months before BMD)	1 (1.6)	106 (9.4)	0.037^b^
Bisphosphonates (during 2 years before BMD)	8 (12.9)	296 (26.2)	0.020^b^
Bisphosphonates (ever)	39 (62.9)	694 (61.4)	0.808^b^
Anticonvulsants (during 2 years before BMD)	4 (6.5)	62 (5.5)	0.745^b^

Abbreviations: BC, breast cancer; BMI, body mass index; BMD, bone mineral density; PTH, parathyroid hormone; VitD, vitamin D.

a Statistical analysis: Student's *t*-test.

bStatistical analysis: *χ*^2^-test.

**Table 2:** Population of patients with osteoporotic fractures—fracture location (*n*=1,193)

*Variable*	*All subjects*	*BC (*n*=62)*	*No BC (*n*=1,131)*	P *value*
Hip fracture (*N*, %)	231 (19.4)	10 (16.1)	221 (19.5)	0.508^a^
Vertebral fracture (*N*, %)	436 (36.5)	20 (32.3)	416 (36.8)	0.471^a^
Distal radius fracture (*N*, %)	241 (20.2)	16 (25.8)	225 (19.9)	0.295^a^
Ribs fracture (*N*, %)	126 (10.6)	11 (17.7)	115 (10.2)	0.059^a^
Humerus fracture (*N*, %)	277 (23.2)	16 (25.8)	261 (23.1)	0.620^a^
Time from BMD to first osteoporotic fracture (median, interquartile range, years)	2.01 (0.1–4.2)	2.14 (0.9–4.5)	2.01 (0.1–4.2)	0.222^b^

Abbreviations: BC, breast cancer; BMD, bone mineral density.

Some patients had more than one fracture type.

a Statistical analysis: *χ*^2^-test.

b Statistical analysis: a parametric test (Mann–Whitney).

**Table 3:** BC patients stratified by osteoporotic fracture occurrence

*Variable*	*Osteoporotic fracture (*n*=62)*	*No osteoporotic fracture (*n*=596)*	P *value*
*a: Baseline characteristics (*n*=658)*			
Age at BC diagnosis (mean±s.d.)	62.6±9.3	60.0±11.1	0.082^a^
BMI (mean±s.d.)	29.3±4.8	29.9±5.8	0.343^a^

*BMI,* N (%)			
⩽30	39 (62.9)	337 (56.5)	0.442^b^
30.1–35	15 (24.2)	147 (24.7)	
35.1–40	7 (11.3)	73 (12.2)	
⩾40.1	1 (1.6)	39 (6.5)	
HRT treatment, *N* (%) (*n*=296) per history	6 (25.0) (*n*=24)	71 (26.1) (*n*=272)	0.906^b^
HRT (during 2 years before BMD) according to drug purchase, *N* (%)	13 (21.0)	127 (21.3)	0.950^b^
Past/current oral contraceptives use, *N* (%) (*n*=125)	3 (25.0) (*n*=12)	26 (23.0) (*n*=113)	0.877^b^
			
*b: BC grade, stage, and treatment*			
*T stage,* N *(%) (*n=*638)*			
T *in situ*	7 (11.9)	48 (8.3)	0.394^b^
T0	0 (0)	7 (1.2)	
T1	39 (66.1)	331 (57.2)	
T2	12 (20.3)	160 (27.6)	
T3	0 (0)	21 (3.6)	
T4	0 (0)	6 (1.0)	
Tx	1 (1.7)	6 (1.0)	
			
*Stage,* N *(%) (*n*=636)*			
0/I	37 (63.8)	302 (52.2)	0.038^b^
II	21 (36.2)	225 (38.9)	
III/IV	0 (0)	51 (8.8)	

*Histology,* N *(%) (*n*=639)*			
DCIS (only)	7 (11.9)	48 (8.3)	0.467^b^
Invasive duct carcinoma	47 (79.7)	468 (80.7)	
Invasive lobular carci	2 (3.4)	41 (7.1)	
Other	3 (5.1)	24 (4.2)	
			
*Histological grade,* N *(%) (*n*=429)*			
Low	6 (14.0)	106 (27.5)	0.048^b^
Intermediate	25 (58.1)	154 (39.9)	
High	12 (27.9)	126 (32.6)	
			
*Immunohistochemistry,* N *(%)*			
ER positive (*n*=562)	46 (86.8)	426 (83.7)	0.558^b^
PR positive (*n*=552)	42 (80.8)	352 (70.4)	0.115^b^
Her-2 (*n*=436)			
0/+1	30 (76.9)	313 (78.8)	0.192^b^
+2 and CISH negative	0 (0)	12 (3.0)	
+2 and CISH unknown	6 (15.4)	28 (7.1)	
+3/CISH positive	3 (7.7)	44 (11.1)	
Triple negative (*n*=526)	1 (2.0)	28 (5.9)	0.253^b^
Chemotherapy (neo or adjuvant) *N* (%)(*n*=636)	15 (25.0)	260 (45.1)	0.003^b^

*Hormonal therapy (neo or adjuvant) (*n*=636)* N *(%)*			
Tamoxifen alone	31 (51.7)	198 (34.4)	0.007^b^
Aromatase inhibitors alone	1 (1.7)	46 (8.0)	
Tamoxifen+aromatase inhibitors	20 (33.3)	206 (35.8)	
Other	1 (1.7)	1 (0.2)	
Any tamoxifen treatment, *N* (%)	51 (85.0)	404 (70.1)	0.015^b^
Any aromatase inhibitors treatment, *N* (%)	21 (35.0)	252 (43.8)	0.193^b^
Radiotherapy (neo or adjuvant), *N* (%) (*n*=636)	42 (70.0)	440 (76.4)	0.272^b^
Trastuzumab (neo or adjuvant), *N* (%) (*n*=635)	0 (0)	16 (2.8)	0.195^b^
			
*c: BMD*			
*BMD, g/cm*^*2*^ *(mean*±*s.d.)*			
Femoral neck	0.77±0.11	0.83±0.12	<0.001^a^
Total hip	0.84±0.13	0.91±0.14	<0.001^a^
Spine	0.96±0.15	1.03±0.17	0.002^a^

*BMD* T-*score (mean*±*s.d.)*			
Femoral neck	−1.72±0.94	−1.22±1.04	<0.001^a^
Total hip	−1.32±1.12	−0.79±1.13	<0.001^a^
Spine	−1.80±1.23	−1.23±1.42	0.002^a^

*BMD* Z-*score (mean*±*s.d.)*			
Femoral neck	−0.37±0.84	−0.05±0.93	0.010^a^
Total hip	−0.16±1.03	0.18±1.02	0.013^a^
Spine	−0.29±1.34	0.13±1.49	0.035^a^

Abbreviations: BC, breast cancer; BMD, bone mineral density; BMI, body mass index; CISH, chromogenic *in situ* hybridization; DCIS, ductal carcinoma *in situ*; ER, estrogen receptor; HRT, hormone replacement therapy; PR, progesterone receptor.

a Statistical analysis: Student's *t*-test.

b Statistical analysis: *χ*^2^-test.

**Table 4:** Multivariable analysis (Cox regression) to first osteoporotic fracture

*Variable*	*HR*	*95% CI*	*HR*	P *value*
Age at BMD	1.02	1.02–1.03	1.02	<0.001
BMI	1.03	1.02–1.04	1.03	<0.001

*BMD* T-*score*				
Total hip	0.71	0.66–0.76	0.71	<0.001
Spine	0.94	0.89–0.99	0.94	0.014
BC	1.34	1.04–1.73	1.34	0.026

Abbreviations: BC, breast cancer; BMI, body mass index; BMD, bone mineral density; CI, confidence interval; HR, hazard ratio.
